# Towards elimination of hepatitis B and C in European Union and European Economic Area countries: monitoring the World Health Organization’s global health sector strategy core indicators and scaling up key interventions

**DOI:** 10.2807/1560-7917.ES.2017.22.9.30476

**Published:** 2017-03-02

**Authors:** Erika F Duffell, Dagmar Hedrich, Otilia Mardh, Antons Mozalevskis

**Affiliations:** 1 European Centre for Disease Prevention and Control (ECDC), Stockholm, Sweden; 2 European Monitoring Centre for Drugs and Drug Addiction (EMCDDA), Lisbon, Portugal; 3 WHO Regional Office for Europe, Copenhagen, Denmark

**Keywords:** Keywords: hepatitis B, hepatitis B virus, hepatitis C, hepatitis C virus, viral hepatitis, public health policy, prevention, elimination, monitoring, indicators, strategy, people who inject drugs, injecting drug use

## Abstract

The World Health Organization ‘Global Health Sector Strategy on Viral Hepatitis 2016–2021’ aimed at the elimination of viral hepatitis as a public health threat provides a significant opportunity to increase efforts for tackling the epidemics of hepatitis B and hepatitis C virus infections across Europe. To support the implementation and monitoring of this strategy, core epidemiological and programmatic indicators have been proposed necessitating specific surveys, the systematic collection of programmatic data and the establishment of monitoring across the care pathway. European Union and European Economic Area countries already made progress in recent years implementing primary and secondary prevention measures. Indeed, harm reduction measures among people who inject drugs reach many of those who need them and most countries have a universal hepatitis B vaccination programme with high coverage above 95%. However, while a further scaling up of prevention interventions will impact on incidence of new infections, treating those already infected is necessary to achieve reductions in mortality. The epidemiological, demographic and socio-political situation in Europe is complex, and considerable diversity in the programmatic responses to the hepatitis epidemic exists. Comprehension of such issues alongside collaboration between key organisations and countries will underpin any chance of successfully eliminating hepatitis.

## Background

It is estimated that ca 4.7 million people living in European Union (EU) and European Economic Area (EEA) countries are chronically infected with the hepatitis B virus (HBV) and 5.6 million have been infected with the hepatitis C virus (HCV). Both are major causes of chronic liver disease, liver cirrhosis and hepatocellular carcinoma [1]. The resulting burden of disease presents a public health challenge for national health systems. While the incidence of new infections has declined in many European countries due to implementation of effective vaccination programmes (against hepatitis B) and prevention strategies targeting transmission through injecting drug use and healthcare, modelling suggests that morbidity and mortality will continue to increase [2,3]. Indeed, deaths from hepatitis now exceed those from HIV and tuberculosis combined and latest published estimates show that 96,000 people die each year in EU/EEA countries from HBV and HCV-related liver disease [4].

In May 2016, the World Health Assembly adopted the first ‘Global Health Sector Strategy (GHSS) on Viral Hepatitis’ aimed at eliminating viral hepatitis as public health threat [5]. The concept of elimination for these infections is based on reducing the incidence of chronic infections and the associated mortality, with the World Health Organization (WHO) setting global targets for reducing the incidence of chronic infections by 90% and mortality by 65% by 2030. Achieving these targets will require significant scaling-up of key interventions, including hepatitis B childhood vaccination, birth-dose vaccination or other means to prevent mother-to-child transmission, improved systems to assure safe blood transfusions/blood products, injection safety, interventions aimed at preventing transmission among people who inject drugs, and increased testing with linkage to care and treatment.

To support the implementation and monitoring of this strategy, a framework with 10 core indicators has been proposed by WHO, which include a mix of epidemiological and programmatic indicators (Table) [6].

**Table:** Core indicators for the World Health Organization’s monitoring and evaluation framework for hepatitis B and hepatitis C virus elimination 2016–2021

Indicator number	Indicator name
**C1**	Prevalence of chronic HBV and HCV infection
**C2**	Infrastructure for HBV and HCV testing
**C3**	a. Coverage of timely hepatitis B vaccine birth dose (within 24 hours) and other interventions to prevent mother-to-child transmission of HBV b. Coverage of third-dose hepatitis B vaccine among infants
**C4**	Needle–syringe distribution
**C5**	Facility level injection safety
**C6**	People living with HCV and/or HBV diagnosed
**C7**	a. Treatment coverage for hepatitis B patients b. Treatment initiation for hepatitis C patients
**C8**	a. Viral suppression for chronic hepatitis B patients treated b. Cure for chronic hepatitis C patients treated
**C9**	a. Cumulated incidence of HBV infection in children 5 years of age b. Incidence of HCV infection
**C10**	Deaths from hepatocellular carcinoma, cirrhosis and liver diseases attributable to HBV and HCV infection

HBV: hepatitis B virus; HCV: hepatitis C virus.

Source: [6].

The process and criteria for selecting the indicators are described in detail in the WHO technical report [6]. In this paper we provide an overview of the current situation across EU/EEA countries in the context of the global WHO indicators to highlight gaps in programmatic responses and challenges in achieving elimination in Europe.

## The European situation

The WHO Regional Office for Europe (WHO/Europe), in consultation with the Member States and partner organisations, has developed an action plan to guide the implementation of the GHSS in the European Region [7]. This regional plan was launched following endorsement by the Regional Committee in September 2016 and provides the structural framework for countries to use when organising their responses. It includes regional targets, some of which are more ambitious than the global targets in recognition of already existing prevention and control efforts in the Region and the capacity of existing systems to further impact on the epidemics. The plan refers to the WHO monitoring and evaluation framework with 10 core indicators as a tool intended to facilitate the generation, collection and analysis of standardised data for the monitoring of the response on the national and Regional level (Table).

The European Centre for Disease Prevention and Control (ECDC) and the European Monitoring Centre for Drugs and Drug Addiction (EMCDDA), both EU agencies, are well placed to provide technical support to assist EU/EEA countries develop tailored national plans for achieving the WHO targets. In 2016, the two agencies, in collaboration with WHO/Europe, assessed the availability of data for each of the core indicators and concluded that current data sources in most EU/EEA countries are insufficient, particularly for assessing the epidemiological burden and for monitoring the different steps along the cascade of care [8]. Further collaboration with the countries and clinical associations will be required to improve data sources. Regular seroprevalence surveys and sentinel-site surveys will be required to determine (i) estimates of prevalence and incidence, (ii) the attributable fraction of liver cirrhosis and hepatocellular carcinoma cases related to HBV and HCV infections and (iii) the size of the undiagnosed population [6]. The systematic collection of programmatic data related to testing and to prevention and treatment coverage will also need to be conducted.

While some EU/EEA countries have well-developed data systems providing comprehensive epidemiological information on hepatitis B and C to support local policy initiatives, there is variation between countries [9]. In an attempt to address such differences and standardise notification data, ECDC implemented in 2011 an enhanced surveillance system to facilitate the collection of data on newly diagnosed cases. Recognising the limitations of routine notification data to provide a clear epidemiological overview of the numbers and groups affected by infection, EMCDDA and ECDC have started work to collect and collate seroprevalence data from key risk groups and the general population using standardised methodologies and will publish this information when available.

From an epidemiological perspective, the prevalence of HBV and HCV is low-to-intermediate in most EU/EEA countries, but the situation is diverse and dynamic. National estimates of seroprevalence in the general population vary from 0.1% to 4.4% for HBV and from 0.1% to 5.9% for HCV [1]. Among key risk groups, prevalence estimates show similar variation. For the population of people who inject drugs (PWIDs) and former PWIDs in Europe, the prevalence of HCV is high, with 11 of the 16 countries with recent data reporting national estimates of over 40% [1]. Harm reduction programmes, especially those combining needle and syringe programmes (NSP) and opioid substitution treatment of people who inject opioids, as well as more recently, treatment with the new direct-acting antiviral drugs, may have the potential to contribute considerably to reducing transmission in many countries. In spite of this, prevalence rates found in national and subnational seroprevalence studies among PWID in most EU/EEA countries are high (> 50%) [10], including among young and new injectors [11]. Reports suggest that only a small proportion of those infected with HBV or HCV are aware of their infection [2,12]. Among PWIDs, the proportion of those undiagnosed for HCV is likely to be very high, with estimates ranging from 24% to 76% [13]. This highlights a clear need to extend existing testing programmes.

Migrants, defined as individuals born outside their country of residence, contribute to the HBV and HCV prevalence pool. A recent analysis estimated that 1 to 2 million chronically HBV-infected migrants from endemic countries with a prevalence of over  2%, reside in the EU/EEA and account for 25% of all chronic HBV cases [14]. For HCV, estimates indicate that chronic infections among migrants account for 14% of all chronic infections [14].

Men who have sex with men (MSM) are a key risk group for current HBV and HCV transmission in most European countries. Vaccination has reduced HBV transmission, however, there have been increasing reports from European countries of acute HCV infections among HIV-infected MSM [15]. Reports of HCV infections among HIV-negative MSM have raised concern that HCV is an expanding epidemic among MSM [1].

Despite the emerging trends described above and high levels of infection among key risk groups, the incidence of HBV and HCV has declined slightly across Europe in recent years [2,12]. For HBV, this is demonstrated by the surveillance data reported to ECDC which have shown a steady decline in the rates of acute infections across EU/EEA countries, with rates in most countries now less than 1 case per 100,000 [16]. However, there remains considerable diversity between countries with notification rates for acute HBV cases in 2014 ranging from 0 cases in Malta to 3.2 per 100,000 in Bulgaria. While chronic viral hepatitis is known to be one of the leading causes of end-stage liver disease, estimation of the proportion of deaths from liver cirrhosis and hepatocellular carcinoma attributable to HBV and HCV infection is difficult due to scarcity of data [17].

Data on hepatitis B vaccination coverage are routinely collected by WHO and the United Nations Children's Fund (UNICEF) through Joint Reporting Form on Immunization [18]. Twenty-three of the 31 EU/EEA countries reported data on coverage with three doses of HBV vaccine among 1-year-olds in 2014. Of these 23 countries, 11 reported coverage of 95% or over [18]. EU/EEA countries offer the first dose at birth either as a general recommendation to all newborns (7/31) or targeted to newborns from mothers from groups at risk or mothers with HBV infection (24/31) [19].

In relation to the indicator on injection safety, there is no systematic data collection of facility level injection safety in EU/EEA countries, but evidence from the notification data submitted to ECDC indicates that nosocomial transmission remains an ongoing transmission route for both infections in some countries [16,20].

Data on the levels of testing and treatment in EU/EEA countries are currently not systematically collected at the EU/EEA level or even nationally in most countries, but the available published evidence of ad hoc reviews suggests that provision is suboptimal in many countries, with high numbers of infections undiagnosed and only a small proportion of those who have been diagnosed effectively treated [13,21].

Programmatic data relating to prevention programmes for HBV and HCV across EU/EEA countries, although incomplete, show similar levels of diversity. The data collected by EMCDDA on harm reduction measures targeting injecting drug users show considerable variation across the region with suboptimal levels in many countries. Indeed, while the data indicate that one in two problem opioid users in Europe receive opioid substitution treatment (OST), in some countries the fraction of high-risk opioid users receiving OST is less than 20% [10]. In 14 countries providing recent estimates of the size of the PWID population, the number of syringes distributed per year from specialised NSPs remains below 50 syringes per injector in three countries and only four countries were able to document coverage above the recommended threshold of 200 syringes/PWID/year [10].

In addition to current gaps in prevention programmes and the available data required to monitor the implementation of these programmes, EU/EEA countries face other challenges to the successful elimination of hepatitis B and C. While recent data indicate that injecting drug use is stable or declining in Europe, the prevalence of injecting drug use ranges between 1 and 9 cases per 1,000 population aged 15-64 years and is high (> 4 /1,000) in five countries [22]. Furthermore, a potentially large population of HCV infected ex-injectors might need to be included in future healthcare estimates [11].

The population of migrants coming from countries with high endemicity for HBV and HCV is dynamic and recent studies indicate that estimates of prevalence from the country of origin may not be a good proxy for prevalence in all migrant groups. The prevalence in migrant populations has been found to be lower, especially for hepatitis B, so the true extent of the burden among different migrant groups is unclear [14].

Interventions are further hampered as stigma and discrimination surround hepatitis B and C, migrants, MSM and injecting drug use. In some parts of eastern Europe, repression is the prevailing response to drug use, while across most of the EU, a balanced approach with public health and criminal justice elements is now common [23-25]. Indeed, stigma and discrimination are barriers to testing and treatment access among PWID. Stigma around hepatitis B infection has been shown to impact negatively on testing behaviour of some migrant groups [26].

The EU/EEA is mostly comprised of high income countries. However, resources dedicated to the prevention and control of hepatitis have been described as suboptimal [21] and in striving towards elimination and the necessary scaling up of services, this will need to be addressed. The current cost of antiviral drugs for curing hepatitis C remains high and this could undermine national efforts in impacting upon the growing disease burden. Indeed, while prevention measures are able to impact on the incidence of new infections [13,16], it is only through identifying and treating those already infected that a reduction in mortality will be possible. EU mechanisms such as the joint procurement of medical countermeasures [27] could be one option for countries to consider, to help reduce the costs of antiviral treatment, while continued advocacy by non-governmental organisations remains important. WHO has developed several tools to assist countries in their prevention and control efforts including global testing and treatment guidance and national planning toolkits [28-30]. ECDC and EMCDDA provide complementary tools, such as specific evidence-based recommendations for action, tailored to the EU context, and both agencies will continue to work in close collaboration with WHO to support countries in their efforts to scale up activities.

Further development of existing monitoring platforms and working to minimise the reporting burden for countries is important and prevention and control efforts for hepatitis could benefit from understanding some of the lessons learnt in relation to HIV in this area. Indeed, developing a standardised monitoring approach for interventions including diagnosis and treatment along the continuum of care, which is already established for HIV, could now be considered for hepatitis B and C. A recent review of operational interventions along the chronic viral hepatitis care continuum for people with diagnosed or undiagnosed chronic viral hepatitis demonstrated that a range of relatively simple, inexpensive operational interventions can substantially improve engagement and retention along the cascade of care, thereby optimising the implementation of screening, care, and treatment programmes [31].

## Conclusions

The launch of a global strategy aimed at the elimination of viral hepatitis provides an opportunity to increase efforts aimed at tackling the HBV and HCV epidemics. European countries have already made progress in recent years implementing primary and secondary prevention measures. Indeed, measures aimed at reducing health-related harm among PWIDs, such as OST and NSP, now reach many of those who need them and most countries have in place a hepatitis B vaccination programme with high levels of coverage. These measures have had an impact on the epidemiology of HBV and HCV. However, the epidemiological, demographic and socio-political situation is complex in Europe and diversity and inequities in the programmatic responses to the epidemics exist. Stigma and discrimination are both important in Europe in relation to hepatitis B and C and efforts to reducing or eliminating stigma are essential if disease elimination is to be achieved. Comprehension of such issues alongside collaboration between key organisations and countries will underpin any chance of successfully eliminating hepatitis.
